# Bibliography

**Published:** 1895-12

**Authors:** 


### Bibliography.

Methoden und Neuerungen auf dem Gebiete der Zahnheilkunde. Von Wilh. Herbst, Zahnarzt in Bremen. Verlag: Odontologische Verlagsanstalt. Berlin.

This work of Dr. Herbst is the result of twenty-five years of practice, as stated in his preface, and covers the public descriptions given of his original methods in this and other countries. Those who recall the author during his visit to the United States will renew the interest then felt in this more detailed account of his ingenious appliances.

Whatever may have been thought of his rotation method of filling teeth, whether for or against, great credit was due him for his untiring efforts to explain, as far as one unacquainted with English could explain, the value of this original idea. The impression he left upon American dentistry was of great value, as his work demonstrated to astonished audiences what could be done by an inventive and energetic mind outside of schools and largely isolated from the active dental world. Since then this same creative force has evolved many original ideas. These he has given in this book of 295 pages, extensively illustrated.

The first part very naturally begins with the work that made his name famous,—"Gold Filling after Herbst's Method of Rotation." This chapter covers the whole process, with full illustration of his various matrices. Most of these are familiar to those who saw him work.

His method of preparing glass inlays is described, followed in the second part by crown- and bridge-work treated in detail.

The third part is largely made up of novelties in practical work. The last chapter is devoted to the manufacture of gold for dental purposes.

This book has a value peculiarly its own, and while it is doubtful whether, if translated, it would add materially to our practical knowledge on operative or mechanical work, it would still be gratifying to many English readers to have Dr. Herbst's methods in permanent form and of easy reference.

